# Virtual (Self) Reflection: Frequent Videoconferencing Usage Is Uniquely Associated With Body Dissatisfaction and Dietary Restraint Symptoms Among Adults

**DOI:** 10.1002/erv.3191

**Published:** 2025-03-21

**Authors:** Jade Portingale, Isabel Krug

**Affiliations:** ^1^ Melbourne School of Psychological Sciences The University of Melbourne Melbourne Australia

**Keywords:** body image, disordered eating, eating disorders, mood, rejection sensitivity, screen‐time, videoconferencing

## Abstract

**Objective:**

This study examined the relationship between videoconferencing usage frequency for work/study purposes and eating disorder (ED)‐related symptoms, focusing on psychological factors that may heighten vulnerability to such concerns in appearance‐focused interactions.

**Method:**

Australia‐based participants (*N* = 1820; 76% female; *M*age = 20.28, *SD* = 4.43) completed an online survey assessing videoconferencing usage frequency for work/study, ED‐related symptoms (body dissatisfaction, disordered eating [DE], depression), and psychological factors (appearance‐based rejection sensitivity [appearance‐RS], self‐objectification, body‐ideal internalization). Participants were categorized as higher‐frequency users (multiple times per week or more; *n* = 1334; 73%) or lower‐frequency users (once per week or less; *n* = 486; 27%).

**Results:**

Body‐ideal internalization and appearance‐RS showed small to medium positive associations with all ED‐related symptoms. Higher‐frequency users reported greater body dissatisfaction and DE symptoms (overall, dieting, and oral control), though these effects were modest, explaining minimal variance beyond established risk factors. Videoconferencing frequency was unrelated to depressive or bulimia/food preoccupation symptoms and rarely moderated psychological factor‐symptom relationships, with three exceptions: higher frequency usage modestly weakened the positive association between appearance‐RS and DE (overall and oral control) and modestly strengthened the positive association between self‐objectification and buimia/food preoccupation DE symptoms.

**Conclusions:**

These findings suggest that frequent work/study‐related videoconferencing may be uniquely associated with body dissatisfaction and dietary restraint symptoms, particularly for those with a tendency to self‐objectify. Future research into individual differences, usage contexts, and face‐related outcomes is warranted.

**Summary**

- Appearance‐based rejection sensitivity (appearance‐RS) and body ideal internalization positively associated with eating disorder symptoms; self‐objectification showed no association.
- Higher videoconferencing frequency associated with slightly higher body dissatisfaction and disordered eating (DE) symptoms related to dieting and oral control; unrelated to depression or bulimia/food preoccupation.
- Higher videoconferencing frequency modestly strengthened the link between appearance‐RS and self‐objectification with specific DE symptoms.

## 1 Introduction

The coronavirus disease 2019 (COVID‐19) pandemic accelerated reliance on videoconferencing platforms for work and study, surpassing in‐person meetings (Iqbal 2021). Even today, videoconferencing remains a common interaction tool (Pires 2024). Unlike traditional meetings, videoconferencing exposes users to a video of their own image, particularly their face, throughout the meeting (Choukas‐Bradley et al. 2022). This setup resembles prolonged mirror‐gazing (Jarman et al. 2023; Pfund et al. 2020; Pikoos et al. 2021), which research suggests can elevate distress, body dissatisfaction, and self‐critique among healthy individuals (Shafran et al. 2007; Veale et al. 2016).

Videoconferencing environments may prompt users to modify their appearance or avoid using cameras altogether (Sarangi et al. 2022), with appearance concerns cited as the primary reason for disabling cameras during calls (Castelli and Sarvary 2021). These behaviors resemble body checking and avoidance, which are linked to body image concerns and eating disorders (EDs) (Fairburn et al. 2003; Nikodijevic et al. 2018). Features such as gallery view may further amplify appearance scrutiny and comparisons, heightening body image and eating concerns (Jarman et al. 2023; Pfund et al. 2020; Pikoos et al. 2021).

### 1.1 Support for the Relationship Between Videoconferencing Use, Disordered Eating Symptoms and Correlates

Several studies have linked videoconferencing use to body image concerns, eating disturbance and mood concerns during and after the COVID‐19 pandemic using trait‐based measures (Chen et al. 2021; Choukas‐Bradley et al. 2022; Cristel et al. 2020; Gullo and Walker 2021; Hart et al. 2023; Harriger and Pfund 2022; Jarman et al. 2023; Pfund et al. 2020; Pikoos et al. 2021; Stewart and Stapleton 2023). A review by Hart et al. (2022) found consistent associations between self‐view exposure and increased appearance concerns (e.g., dissatisfaction) and appearance‐management behaviors, particularly among ED at‐risk individuals. However, methodological limitations and gaps remain.

Research on videoconferencing, eating pathology, and mood is particularly sparse, with mixed findings. Regarding eating pathology, Hart et al. (2023) reported positive correlations between ED risk (*n* = 245 at‐risk) and problematic videoconferencing behaviors in a community sample, however, relied on the SCOFF (Morgan et al. 1999) screening tool, which has low specificity in community samples (Solmi et al. 2015). While Gullo and Walker (2021) found no association with binge eating symptoms, they aggregated videoconferencing use with other screen‐based activities (e.g., selfie‐taking) and assessed only one aspect of eating pathology. Regarding mood, two studies revealed positive correlations between videoconferencing use and depressive symptoms over the past 2 weeks (Choukas‐Bradley et al. 2022; Gullo and Walker 2021). A recent ecological momentary assessment (EMA) study by Portingale, Kenny, et al. (2024), and the only study to examine state‐level relationships, found that videoconferencing use predicted exercise urges, but not urges for restrictive eating or overeating, or mood, at the state level. While valuable for understanding immediate contextual effects, this state‐level analysis may not capture enduring symptoms or behaviors that may emerge from sustained videoconferencing exposure.

Additionally, studies have rarely examined videoconferencing usage frequency specifically for work/study, despite this being a primary context for mandatory and sustained videoconferencing engagement, particularly during the COVID‐19 pandemic (Karl et al. 2022). Theoretically, appearance‐related concerns in professional/academic contexts may be particularly impactful due to the combination of evaluative pressure, performance expectations, and mandatory sustained exposure to one's self‐image. Some studies have assessed videoconferencing use for work/study (Hart et al. 2023; Jarman et al. 2023; Portingale, Kenny, et al. 2024), but overlooked the impact of usage frequency, despite the frequency of digital media use being central to understanding its psychological impact (Salomon and Brown 2019; Twenge and Martin 2020). Stewart and Stapleton (2023) provided initial evidence that work/study‐related videoconferencing frequency, but not social usage, significantly predicts body image concerns. However, their study failed to examine whether these effects extend to eating pathology and mood, and investigate the mechanisms underlying this relationship.

Indeed, no study has concurrently examined body dissatisfaction, DE symptoms, and depressive symptoms in relation to videoconferencing use, despite being theoretically interconnected. Additionally, most studies overlooked key ED‐related psychological factors that have established roles in appearance‐focused social interactions, such as self‐objectification (Schaefer et al. 2018), body‐ideal internalization (Keel and Forney 2013), and appearance‐based rejection sensitivity (appearance‐RS) (De Paoli et al. 2017). These factors might explain individual differences in vulnerability to ED‐related symptoms following videoconferencing.

### 1.2 Self‐Objectification, Body‐Ideal Internationalization, and Videoconferencing

Self‐objectification, the tendency to view oneself as an aesthetic object (Quinn et al. 2006), is consistently linked to body dissatisfaction, DE, and depressive symptoms (Jones and Griffiths 2015; Schaefer et al. 2018). Videoconferencing inherently encourages self‐observation, potentially intensifying self‐objectification tendencies. Individuals predisposed to self‐objectification may engage in excessive self‐view checking and appearance adjustments during calls, potentially heightening body image and eating concerns. Pfund et al. (2020) found that self‐objectification moderated the relationship between videoconferencing hours and appearance satisfaction, though its effects on other symptoms remain unclear.

Body‐ideal internalization, which often precedes self‐objectification (Tan et al. 2016), involves adopting societal beauty standards as personal goals (Harrison and Hefner 2006) and is consistently implicated in body dissatisfaction, DE, and depressive symptoms (Tylka 2011; Keel and Forney 2013). Studies using related technologies have linked selfie‐taking and editing to increased thin‐ideal internalization and body dissatisfaction (McGovern et al. 2022). Since videoconferencing platforms enable constant appearance monitoring and editing (e.g., using face filters), high body‐ideal internalization may increase vulnerability to body image and eating concerns.

### 1.3 Appearance‐Based Rejection Sensitivity and Videoconferencing

Appearance‐RS—anxious expectations of rejection based on physical appearance (Park 2007)—is also linked to body dissatisfaction, DE and depressive symptoms (Calogero et al. 2010; De Paoli et al. 2017; Park et al. 2010). Though unstudied in videoconferencing contexts, appearance‐RS correlates with social anxiety in online interactions (Papapanou et al. 2023) and greater selfie‐editing behaviors on social media (Hawes et al. 2020). During videoconferencing, appearance‐RS may amplify self‐scrutiny and interpretation of peer or colleague reactions as appearance‐related rejection, exacerbating ED‐related symptoms.

### 1.4 The Current Study

Prior research on the relationship between videoconferencing and ED‐related symptoms has been limited by heterogeneous measures and a lack of comprehensive assessments of DE symptoms, body dissatisfaction, and mood in the same population. Moreover, psychological factors like self‐objectification, body‐ideal internalization, and appearance‐RS remain underexplored in this context, despite their theoretical relevance.

This study aimed to address these gaps by examining: (1) associations between these psychological factors and ED‐related symptoms, and for the first time, whether videoconferencing frequency for work/study (2) correlates with these symptoms and (3) moderates the association between these psychological factors and ED‐related symptoms. We assessed a community‐based sample, recognizing that DE and depressive symptoms exist along a continuum from non‐clinical to clinical levels (Nagl, Hilbert, et al. 2016; Nagl, Jacobi, et al. 2016; Wartberg et al. 2018).

We hypothesized that (1) elevated psychological factors would be associated with greater ED‐related symptoms (H1), (2) higher (vs. lower) videoconferencing usage frequency would be associated with greater ED‐related symptoms (H2), and (3) the relationship between psychological factors and symptoms would be stronger among higher (vs. lower) frequency videoconferencing users (H3).

## 2 Method

### 2.1 Participants

Participants were eligible if they were adults (≥ 18 years) fluent in English. Participants were recruited from across Australia through the Research Experience Programme (REP) at a university in Melbourne and from the general community via online advertisements, university noticeboards, social media posts, snowball sampling, and personal contacts of the researchers. Recruitment occurred during the COVID‐19 pandemic, spanning from April 2020 to October 2022. A total of 1932 participants completed the online survey. Of those, 112 were removed due to duplicate entries or missing baseline data on videoconferencing use. Data quality checks were conducted to identify suspicious response patterns (e.g., long strings of identical responses).

The demographic characteristics of the final sample (*N* = 1820) are displayed in Table 1. Most participants self‐identified as female (76%) and Caucasian (34%) or East/Southeast Asian (30%) and were young adults (*M* = 20.28 years, *SD* = 4.43) with a mean body mass index (BMI) within the ‘healthy’ range. Only 3% of the sample self‐reported a lifetime ED diagnosis. Most participants reported currently using a videoconferencing service for work or study (*n* = 1619; 89%), with Zoom (88%), WhatsApp (20%), and Microsoft Teams (17%) being the most commonly used platforms.

**TABLE 1:** Demographic characteristics of the total sample and by videoconferencing user status.

Demographic variable	Statistics				
	Lower frequency videoconferencing user (*n* = 486, 26.7%)	Higher frequency videoconferencing user (*n* = 1334, 73.3%)	Total (*N* = 1820)	*t*/*χ* ^2^	*p*
Age (*M* ± *SD*)	20.10 ± 5.25	20.34 ± 4.16	20.28 ± 4.43	−1.00	0.316
BMI (*M* ± *SD*)	23.74 ± 8.91	22.23 ± 5.36	22.63 ± 6.52	3.47	**< 0.001**
Gender (*n*, %)				12.84	**0.005**
Female	390 (80.6%)	991 (74.3%)	1381 (75.9%)		
Male	87 (17.9%)	330 (24.7%)	417 (22.9%)		
Other/prefer not to say	9 (1.9%)	13 (1.0%)	22 (1.2%)		
Ethnicity (*n*, %)				31.83	**< 0.001**
Caucasian	195 (40.1%)	421 (31.6%)	616 (33.8%)		
Eastern Asian	108 (22.3%)	445 (33.4%)	553 (30.4%)		
Southern Asian/Southeast Asian	119 (24.5%)	347 (26.0%)	466 (25.6%)		
African American	5 (1.0%)	10 (0.7%)	15 (0.8%)		
Hispanic/Latin American	11 (2.3%)	16 (1.2%)	27 (1.5%)		
Middle Eastern	10 (2.1%)	28 (2.1%)	38 (2.1%)		
Aboriginal	0 (0.0%)	2 (0.1%)	2 (0.1%)		
Other	38 (7.8%)	65 (4.9%)	103 (5.7%)		
Highest education completed (*n*, %)				7.99	0.239
Year 12 or below	85 (17.6%)	162 (12.1%)	247 (13.6%)		
Certificate/diploma	92 (19.0%)	289 (21.7%)	381 (20.9%)		
Bachelor's degree	199 (41.1%)	573 (43.0%)	772 (42.4%)		
Postgraduate degree	108 (22.3%)	310 (23.2%)	418 (23.0%)		
Primary language (*n*, %)				31.81	**< 0.001**
English	334 (68.7%)	722 (54.1%)	1056 (58.0%)		
Other	152 (31.3%)	612 (45.9%)	764 (42.0%)		
Sexual orientation (*n*, %)				12.48	**0.029**
Heterosexual	354 (72.8%)	1038 (77.8%)	1392 (76.5%)		
Homosexual	19 (3.9%)	41 (3.1%)	60 (3.3%)		
Bisexual	83 (17.1%)	176 (13.2%)	259 (14.2%)		
Asexual	6 (1.2%)	16 (1.2%)	22 (1.2%)		
Other	14 (2.9%)	18 (1.3%)	32 (1.8%)		
Prefer not to say	10 (2.1%)	45 (3.4%)	55 (3.0%)		
Marital status (*n*, %)				4.19	0.522
Single	327 (67.3%)	908 (68.1%)	1235 (67.9%)		
Married	10 (2.1%)	35 (2.6%)	45 (2.5%)		
De facto	3 (0.6%)	11 (0.8%)	14 (0.8%)		
Separated/divorced	3 (0.6%)	2 (0.1%)	5 (0.3%)		
In a relationship	142 (29.2%)	377 (28.3%)	519 (28.5%)		
Lifetime eating disorder diagnosis (*n*, %)				2.39	0.122
Yes	15 (3.1%)	25 (1.9%)	50 (2.7%)		
No	469 (96.9%)	1309 (98.1%)	1770 (97.3%)		
Current eating disorder risk (*n*, %) ^				2.02	0.155
High	106 (21.8%)	334 (25.0%)	440 (24.2%)		
Low	380 (78.2%)	1000 (75.0%)	1,380 (75.8%)		
Current videoconferencing use frequency (*n*, %)				1820.0	**< 0.001**
Never	202 (41.6%)	0 (0.0%)	202 (11.1%)		
Once a month	33 (6.8%)	0 (0.0%)	33 (1.8%)		
Multiple times a month	64 (13.2%)	0 (0.0%)	64 (3.5%)		
Once a week	187 (38.5%)	0 (0.0%)	187 (10.3%)		
Multiple times a week	0 (0.0%)	859 (64.4%)	859 (47.2%)		
Once a day	0 (0.0%)	115 (8.6%)	115 (6.3%)		
Multiple times a day	0 (0.0%)	360 (27.0%)	360 (19.8%)		
					

*Note:* Significant *p* values (< .05) bolded. BMI = Body Mass Index (kg/m^2^). *M* = mean, *SD* = standard deviation. *t*‐test for continuous variables, chi‐squared test for categorical variables. ^ Based on EAT‐26 cut‐off scores of ≥ 20 (high risk) and < 20 (low risk) (Garner et al. 1982).

### 2.2 Measures

#### 2.2.1 Demographics

Participants self‐reported information regarding age, current self‐reported height, weight (to calculate BMI; kg/m^2^), ethnicity, primary language, educational attainment, sexual orientation, marital status, current paid employment status, and lifetime ED diagnosis (*no* = 1, *yes* = 2).

#### 2.2.2 Videoconferencing Use

Participants indicated whether they were currently using a videoconferencing service for work or study (*yes/no*). If they answered yes, they specified the platform used (e.g., *Zoom, Microsoft Teams, WhatsApp*) and the frequency of usage on a seven‐point scale (*never, once a month, multiple times a month, once a week, multiple times a week, once a day, multiple times a day*). Participants were categorized as higher‐frequency users (coded as 2) if they reported engaging multiple times a week or more, and lower‐frequency users (coded as 1) if they reported usage once a week or less, including non‐users. This binary classification was adopted due to the non‐equal intervals between the original scale categories and to facilitate practical interpretation of usage patterns in work/educational settings. Further supporting a binary categorization is research suggesting that appearance‐related technology use may have threshold effects rather than linear relationships with appearance‐based psychological outcomes (Marengo et al. 2018; Salomon and Brown 2019).

While standardized measures of videoconferencing frequency do not yet exist, our chosen cut‐point reflects a meaningful distinction between usage patterns where videoconferencing is integrated into weekly routines versus serving as an occasional tool. This aligns with emerging evidence that higher weekly videoconferencing engagement specifically predicts increased body image concerns (Stewart and Stapleton 2023), though optimal frequency thresholds remain to be established.

#### 2.2.3 Body Ideal Internalization

The Sociocultural Attitudes Toward Appearance Questionnaire (SATAQ‐4; Schaefer et al. 2015) assessed the internalization of appearance ideals (thin/low body fat and muscular/athletic ideals) across 10 items (e.g., “*It is important for me to look athletic*”) rated on a five‐point Likert scale (1 = *definitely disagree*; 5 = *definitely agree*). Items were totaled (range: 10–50), where higher scores indicate greater body‐image‐ideal internalization. Internal consistency for this scale was good in the present study (Cronbach's alpha = 0.85).

#### 2.2.4 Self‐Objectification

Self‐objectification was assessed using the Self‐Objectification Questionnaire (SOQ; Noll and Fredrickson 1998). Participants rank‐ordered 12 body attributes—six appearance‐based (e.g., weight, sex appeal, physical attractiveness) and six competence‐based (e.g., health, strength, energy level)—based on their importance to their physical self‐concept from 1 (*least impact*) to 12 (*most impact*). Self‐objectification scores were calculated by subtracting total competence‐based scores from total appearance‐based scores (range: −36 to +36), where higher scores indicate a greater reliance on appearance for self‐concept. While traditional internal consistency metrics are inappropriate for this rank‐ordered measure, the SOQ has demonstrated satisfactory construct validity and test‐retest reliability (Hu et al. 2025; Noll 1997).

#### 2.2.5 Appearance‐Based Rejection Sensitivity

Appearance‐RS was assessed using the shortened version of the appearance‐RS scale (Park 2007). The scale presented 10 hypothetical scenarios (e.g., *“You are leaving your house to go on a first date when you notice a blemish on your face*”) for which participants indicated, on a 6‐point scale, their anxiety about being rejected (*1 = very unconcerned, 6* = *very concerned*), and expectation of rejection (*1 = very unlikely, 6 = very likely*). For each scenario, anxiety scores were multiplied by expectation scores, which were then averaged across scenarios; leading to a mean score (range: 1–36). Higher scores indicate greater appearance‐RS. Internal consistency was excellent in the present study (Cronbach's alpha = 0.95).

#### 2.2.6 Body Dissatisfaction

The shortened Body Shape Questionnaire (Cooper et al. 1987; Evans and Dolan 1993) measured participants' body weight and shape concerns across eight items (e.g., “*Have you felt excessively large and rounded*?”) rated on a 6‐point Likert scale (1 = *never*; 6 = *always*). Items were totaled (range of 8–48), where higher scores indicate greater body dissatisfaction. Internal consistency was excellent in the present study (Cronbach's alpha = 0.93).

#### 2.2.7 Depressive Symptoms

The Center for Epidemiologic Studies Short Depression Scale (CES‐D 10; Radloff 1977) assessed the frequency of depression symptomatology over the past week across 10 items (e.g., “*I felt depressed*”) rated on a four‐point Likert scale (0 = *rarely or none of the time*; 4 = *all of the time*). Two items were positive affect statements and reverse‐scored. The total score was calculated (range: 0–40), with higher scores indicating greater depressive symptomatology. Internal consistency for this scale was questionable in the current study (Cronbach's alpha = 0.68), though prior research has observed similar levels in community‐based Australian samples (Cronbach's alpha = 0.70) (Mohebbi et al. 2018).

#### 2.2.8 Disordered Eating Symptoms

The Eating Attitudes Test‐26 (EAT‐26; Garner et al. 1982) measured participants' attitudes, feelings, and behaviors related to DE. The EAT‐26 includes 26 items rated on a 6‐point Likert scale from 0 (never) to 5 (always) across three subscales: Dieting (13 items, e.g., “I am terrified about being overweight”), Bulimia/Food Preoccupation (6 items, e.g., “I find myself preoccupied with food”) and Oral Control (7 items, e.g., “I avoid eating when I am hungry”). Scores are summed to produce a total score (range: 0–78) and three subscale scores (Dieting: 0–39; Bulimia/Food Preoccupation: 0–18; Oral Control: 0–21). Total scores ≥ 20 indicate a high level of ED risk. In the current study, internal consistency was excellent for the total score (Cronbach's alpha = 0.93), good for dieting (*α* = 0.89) and bulimia/food preoccupation (*α* = 0.80), and questionable for oral control (*α* = 0.69). Prior research has reported similar patterns of internal consistency across clinical and non‐clinical populations, with the highest internal consistency for the total score and the Dieting subscale, and lower consistency for the Bulimia/Food Preoccupation and Oral Control subscales (Rivas et al. 2010; Spivak‐Lavi et al. 2023; Siervo et al. 2005).

### 2.3 Procedure

Participants accessed and completed an online survey (approximately 30 min duration) through an electronic link provided in the study advertisement, which assessed demographics, videoconferencing usage, and the aforementioned measures. The current data were part of a larger daily monitoring study and represented the baseline assessment. REP participants received 1.0‐unit of course credit for completing the baseline component, while community participants were entered into a draw for one of five $100 (AUD) e‐gift cards. This study received approval from a University Human Research Ethics Committee and all participants provided informed consent.

### 2.4 Data Analytic Plan

#### 2.4.1 Statistical Analyses

All data preparation and analyses were conducted in SPSS (version 29.0.0.0). To characterize the sample, preliminary independent samples *t*‐tests examining group differences on ED‐related psychological variables and symptoms were conducted. Due to their exploratory nature, these preliminary analyses were evaluated at the traditional significance level (*α* = 0.05).

Hierarchical multiple regressions were used to test the study hypotheses. Sociodemographic and clinical covariates (gender, age, BMI, lifetime ED diagnosis) were entered in Step1 as these represent relatively stable individual characteristics that consistently predict ED‐related outcomes (Barakat et al. 2023; Hyde and Mezulis 2020; Jebeile et al. 2021; Keski‐Rahkonen 2021). Psychological factors (appearance‐RS, self‐objectification, and body‐ideal internationalization) were entered in Step 2 as these represent established trait‐level risk factors that may be activated or amplified by environmental contexts (H1). Videoconferencing usage frequency was entered in Step 3 (H2) as it represents a contextual factor that may influence symptoms beyond pre‐existing individual differences. The interaction terms between psychological factors and videoconferencing usage frequency were entered in Step 4 (H3). Statistical outcomes were body dissatisfaction, DE symptoms (overall, oral control, bulimia/food preoccupation, dieting), and depressive symptoms. To ensure robustness of findings, analyses were also conducted using videoconferencing frequency as a continuous variable (coded 0‐6, with higher scores indicating more frequent usage).

To control for multiple testing, we employed a family‐wise error correction approach. We applied a Bonferroni correction (*α* = 0.013) for the four DE outcomes, and used the traditional significance threshold (*α* = 0.05) for the conceptually distinct outcomes of body dissatisfaction and depressive symptoms. This approach balances Type I error control within related measure families while maintaining appropriate statistical power for independent constructs. Effect sizes for significant variables were calculated using standardized regression coefficients (β) with the following thresholds: small (≥ 0.10), medium (≥ 0.30), and large (≥ 0.50) (Cohen 2013). Additionally, R‐squared values were examined to assess the models explained variance, with thresholds interpreted as small (≥ 0.02), medium (≥ 0.13), and large (≥ 0.26) (Cohen 2013).

#### 2.4.2 Sample Size Calculation

An a priori power analysis for linear multiple regression with 12 tested predictors (corresponding to Step 3 of our hierarchical model) indicated that a sample size of 1820 was sufficient to achieve 80% power to detect a small effect size (*f* ^2^ = 0.02; Cohen 2013) with an alpha level of 0.05.

## 3 Results

### 3.1 Data Cleaning and Preparation

The data were thoroughly checked and conformed to the key assumptions of the general linear model; thus, they were analyzed in their untransformed state. Collinearity was assessed using variance inflation factors, all were below 2.0, indicating that multi‐collinearity was unlikely to pose a problem. Summed variables exhibited no missing data for participants, ensuring no bias due to missing values.

### 3.2 Sample Characteristics

As shown in Table 1, higher‐frequency (*n* = 1,334, 73%) and lower‐frequency videoconferencing (*n* = 486, 27%) users differed significantly across several characteristics, including BMI, gender, ethnicity, primary language, and sexual orientation. Higher‐frequency users were more likely to have a lower BMI, identify as male, report Asian ethnicity, and speak a language other than English. No significant between‐group differences were observed for lifetime ED diagnosis or current ED risk.

### 3.3 Descriptive Statistics: Differences Between Higher‐ and Lower‐Frequency Videoconferencing Users on Eating Disorder‐Related Psychological Factors and Symptoms

Table 2 reveals that significant group differences were found between higher‐frequency and lower‐frequency videoconferencing users on DE symptoms (overall, oral control, and dieting), which were greater in higher‐frequency users. No group differences were found for the remaining ED‐related symptoms (bulimia/food preoccupation DE symptoms, body dissatisfaction, depressive symptoms), or for any psychological factors.

**TABLE 2:** Differences between higher and lower frequency videoconferencing users on trait‐based variables included in the regression models.

Trait‐based variables	Statistics			
	Lower frequency videoconferencing user, *M* ± *SD*	Higher frequency videoconferencing user, *M* ± *SD*	*t*	*p*
Self‐objectification	0.89 ± 18.61	1.11 ± 18.13	−0.24	0.814
Body‐ideal internalization	30.38 ± 7.94	30.87 ± 8.33	−1.127	0.260
Appearance‐RS	17.34 ± 8.56	16.95 ± 8.47	0.847	0.397
Body dissatisfaction	25.92 ± 10.51	26.30 ± 10.97	−0.665	0.506
DE (overall)	11.78 ± 12.18	13.17 ± 12.85	2.050	**0.041**
DE (oral control)	1.85 ± 2.49	2.19 ± 2.83	−2.448	**0.015**
DE (bulimia/food preoccupation)	2.71 ± 3.25	2.86 ± 3.26	−0.832	0.406
DE (dieting)	6.74 ± 8.06	7.63 ± 8.49	−1.999	**0.046**
Depressive symptoms	12.17 ± 5.94	12.39 ± 5.79	−0.704	0.482

*Note:* Significant *p* values (< .05) bolded (two‐tailed). *t* *=* independent samples *t*‐test. *M* = mean, *SD* = standard deviation. Appearance‐RS = appearance‐based rejection sensitivity. DE = disordered eating.

### 3.4 Relationship Between Videoconferencing Usage Frequency, Eating Disorder‐Related Psychological Factors and Symptoms (H1–H2)

Table 3 presents the associations between ED‐related psychological factors (H1; Step 2) and videoconferencing usage frequency (H2; Step 3) with body dissatisfaction, DE symptoms (overall, oral control, bulimia/food preoccupation, dieting), and depressive symptoms. Body ideal internalization and appearance‐RS showed small to medium positive associations with body dissatisfaction, DE symptoms, and depressive symptoms. Self‐objectification was not significantly related to any of the assessed outcomes. The models explained 47.9% of the variance in body dissatisfaction, 34.5% in dieting DE symptoms, 32.4% in overall DE symptoms (all large effects), 23.1% in bulimia/food pre‐occupation DE symptoms, 20.1% in depressive symptoms (all medium effects), and 7.1% in oral control DE symptoms (small effect). The unique variance attributed to all psychological factors ranged from 3.2% to 38.2% across symptoms.

**TABLE 3:** Hierarchical regression analyses predicting body dissatisfaction, disordered eating symptoms, and depressive symptoms.

	Body dissatisfaction			DE (overall)			DE (oral control)			DE (bulimia/food preoccupation)			DE (dieting)			Depressive symptoms		
	*β*	*t*	*p*	*β*	*t*	*p*	*β*	*t*	*p*	*β*	*t*	*p*	*β*	*t*	*p*	*β*	*t*	*p*
Step 1																		
Age	−0.014	−0.628	0.530	−0.015	−0.658	0.510	−0.054	−2.274	0.023	0.002	0.067	0.947	0.000	0.021	0.983	−0.056	−2.343	**0** **.019**
BMI	0.013	0.549	0.583	0.012	0.506	0.613	0.008	0.332	0.740	−0.013	−0.570	0.569	0.016	0.686	0.493	0.003	0.106	0.916
Lifetime eating disorder diagnosis: no	−0.107	−4.646	< **0** **.001**	−0.268	−11.687	< **0** **.001**	−0.185	−7.780	< **0.001**	−0.281	−12.267	< **0** **.** **001**	−0.227	−9.794	< **0.001**	−0.098	−4.105	< **0** **.001**
Gender: Male	−0.109	−1.180	0.238	0.074	0.796	0.426	0.014	0.151	0.880	0.158	1.713	0.087	0.050	0.531	0.596	−0.361	−3.756	< **0** **.001**
Gender: Female	0.174	1.885	0.060	0.219	2.371	0.018	0.043	0.453	0.650	0.283	3.066	**0.002**	0.203	2.176	**0** **.030**	−0.295	−3.071	**0** **.002**
	*R* ^2^ = 0.097, Δ*R* ^2^ = 0.097, *F* (5, 1733) = 37.400, *p* < **0.001**			*R* ^2^ = 0.101, Δ*R* ^2^ = 0.101, *F* (5, 1733) = 38.893, *p* < **0.001**			*R* ^2^ = 0.039, Δ*R* ^2^ = 0.039, *F* (5, 1733) = 13.939, *p* < **0.001**			*R* ^2^ = 0.102, Δ*R* ^2^ = 0.102, *F* (5, 1733) = 39.553, *p* < **0.001**			*R* ^2^ = 0.082, Δ*R* ^2^ = 0.082, *F* (5, 1733) = 30.930, *p* < **0.001**			*R* ^2^ = 0.027, Δ*R* ^2^ = 0.027, *F* (5, 1733) = 9.436, *p* < 0**.001**		
Step 2																		
Appearance‐RS	0.370	19.167	**< 0.001**	0.283	12.861	**< 0.001**	0.122	4.719	**< 0.001**	0.251	10.671	**< 0.001**	0.280	12.903	**< 0.001**	0.388	16.216	**< 0.001**
Body‐ideal internalization	0.385	20.409	**< 0.001**	0.290	13.517	**< 0.001**	0.084	3.336	**< 0.001**	0.183	7.998	**< 0.001**	0.343	16.232	**< 0.001**	0.077	3.312	**< 0.001**
Self‐objectification	0.013	0.723	0.469	0.024	1.162	0.245	0.035	1.473	0.141	0.013	0.613	0.540	0.018	0.881	0.378	0.009	0.389	0.697
	*R* ^2^ = 0.479, Δ*R* ^2^ = 0.382, *F* (3, 1730) = 423.185, *p* < **0.001**			*R* ^2^ = 0.324, Δ*R* ^2^ = 0.223, *F* (3, 1730) = 190.650, *p* < **0.001**			*R* ^2^ = 0.071, Δ*R* ^2^ = 0.032, *F* (3, 1730) = 20.019, *p* < **0.001**			*R* ^2^ = 0.231, Δ*R* ^2^ = 0.128, *F* (3, 1730) = 96.191, *p* < **0.001**			*R* ^2^ = 0.345, Δ*R* ^2^ = 0.263, *F* (3, 1730) = 231.557, *p* < **0.001**			*R* ^2^ = 0.201, Δ*R* ^2^ = 0.174, *F* (3, 1730) = 125.594, *p* < **0.001**		
Step 3																		
Higher frequency videoconferencing user	0.038	2.175	**0**.**030**	0.060	3.040	**0**.**002**	0.059	2.560	**0.011**	0.036	1.691	0.091	0.056	2.890	**0.004**	0.038	1.742	0.082
	*R* ^2^ = 0.481, Δ*R* ^2^ = 0.001, *F* (1, 1729) = 4.729, *p* = **0.030**			*R* ^2^ = 0.328, Δ*R* ^2^ = 0.004, *F* (1, 1729) = 9.242, *p* = **0.002**			*R* ^2^ = 0.074, Δ*R* ^2^ = 0.004, *F* (1, 1729) = 6.553, *p* = **0.011**			*R* ^2^ = 0.232, Δ*R* ^2^ = 0.001, *F* (1, 1729) = 2.858, *p* = **0.001**			*R* ^2^ = 0.348, Δ*R* ^2^ = 0.003, *F* (1, 1729) = 8.349, *p* = **0.004**			*R* ^2^ = 0.202, Δ*R* ^2^ = 0.001, *F* (1, 1729) = 3.035, *p* = **.082**		
Step 4																		
Higher frequency videoconferencing user*appearance‐RS	−0.163	−1.932	0.053	−0.205	−2.133	**0** **.033**	−0.295	−2.627	**0.009**	−0.187	−1.828	0.068	−0.146	−1.549	0.121	−0.103	−0.983	0.326
Higher frequency videoconferencing user*body‐ideal internalization	0.195	1.867	0.062	−0.060	−0.501	0.616	−0.049	−0.350	0.727	−0.027	−0.215	0.830	−0.064	−0.543	0.587	0.135	1.043	0.297
Higher frequency videoconferencing user*self‐objectification	0.043	0.607	0.544	0.125	1.543	0.123	0.119	1.252	0.211	0.201	2.308	**0** **.021**	0.068	0.849	0.396	0.135	1.521	0.129
	*R* ^2^ = 0.483, Δ*R* ^2^ = 0.002, *F* (3, 1726) = 1.886, *p* = 0.130			*R* ^2^ = 0.331, Δ*R* ^2^ = 0.003, *F* (3, 1726) = 2.353, *p* = 0.070			*R* ^2^ = 0.079, Δ*R* ^2^ = 0.005, *F* (3, 1726) = 2.919, *p* = **0** **.033**			*R* ^2^ = 0.235, Δ*R* ^2^ = 0.003, *F* (3, 1726) = 2.546, *p* = 0.054			*R* ^2^ = 0.350, Δ*R* ^2^ = 0.001, *F* (3, 1726) = 1.247, *p* = 0.291			*R* ^2^ = 0.204, Δ*R* ^2^ = 0.002, *F* (3, 1726) = 1.217, *p* = 0.302		

*Note:* Model one includes demographic and clinical covariates; Model 2 adds ED‐related psychological factors; Model 3 adds videoconferencing frequency; Model 4 adds interaction terms. DE = disordered eating; BMI = body mass index. Reference categories assigned a value of 0: Gender = Other/prefer not to say; Eating disorder = lifetime diagnosis (yes); Videoconferencing user frequency = lower. Significant results are bolded: *p* < 0.05 for body dissatisfaction and depressive symptoms; *p* < 0.013 for disordered eating symptoms (Bonferroni‐corrected for the four DE measures).

Videoconferencing frequency showed positive associations with body dissatisfaction and DE symptoms (overall, oral control, dieting), while no associations were found with bulimia/food preoccupation or depressive symptoms. For all significant relationships, the standardized regression coefficients were below the threshold for small effects. The models explained 48.1% of the variance in body dissatisfaction, 34.8% in dieting DE symptoms, 32.8% in overall DE symptoms (all large effects), and 7.4% in oral control DE symptoms (negligible effect). However, videoconferencing usage frequency accounted for less than 1% of the unique variance in body dissatisfaction and DE symptoms beyond demographic and psychological factors, indicating no substantial contribution.

### 3.5 Moderating Effect of Videoconferencing Usage Frequency (H3)

As shown in Table 3, videoconferencing usage frequency failed to moderate most relationships between ED‐related psychological factors and symptoms (H3; Step 4). However, three exceptions were noted. Higher (vs. lower) frequency usage weakened the positive relationship between (1) appearance‐RS and overall DE symptoms and between (2) appearance‐RS and oral control DE symptoms, whilst strengthening the positive relationship between (3) self‐objectifciation and bullimia/food preoccupation DE symptoms. The significant moderation effects were small and the unique variance attributed to all interaction effects at Step 4 for the model was less than 1%, indicating no substantial contribution.

### 3.6 Covariates

Table 3 (Step 1) also displays the associations between sociodemographic covariates with body dissatisfaction, DE and depressive symptoms. The female gender was positively associated with DE symptoms (overall, bulimia/food preoccupation, dieting), and both female and male genders were negatively associated with depressive symptoms. Endorsing a lifetime ED diagnosis was positively associated with all ED‐related symptoms. Age was negatively associated with oral control DE symptoms and depressive symptoms. BMI was unrelated to the assessed ED‐related symptoms.

Supplementary analyses using videoconferencing frequency as a continuous variable (0–6) yielded consistent patterns of significance, effect size, and variance explained across all outcomes, supporting the robustness of our primary findings using the binary categorization (see Supplementary Material).

## 4 Discussion

We investigated cross‐sectional relationships between videoconferencing usage frequency, ED‐related psychological factors (self‐objectification, body‐ideal internalization, and appearance‐RS), and ED‐related symptoms (body dissatisfaction, DE, and depressive symptoms). Generally consistent with expectations, body‐ideal internalization and appearance‐RS were positively associated with all ED‐related symptoms, while self‐objectification was unrelated. Partially consistent with expectations, higher (vs. lower) videoconferencing usage frequency was positively associated with body dissatisfaction and DE symptoms (overall, oral control, and dieting). However, videoconferencing frequency was unrelated to bulimia/food preoccupation and depressive symptoms. Unexpectedly, videoconferencing frequency failed to moderate most psychological factor‐symptom relationships, with three exceptions, for the relationship between appearance‐RS and DE symptoms (overall and oral control) and between appearance‐RS and bullimia/food preoccupation DE symptoms. Most effects were limited in explanatory power, warranting cautious interpretation.

### 4.1 Positive Associations Between Videoconferencing Usage Frequency, Body Dissatisfaction, and Disordered Eating Symptoms

Higher videoconferencing frequency was associated with greater body dissatisfaction and DE symptoms (overall, oral control, and dieting). These results align with previous research demonstrating positive associations between DE symptoms and problematic videoconferencing behaviors (Hart et al. 2023), and EMA findings that videoconferencing usage predicted greater exercise urges (Portingale, Kenny, et al. 2024). Given that videoconferencing usage frequency contributed minimally to the variance in body dissatisfaction and DE symptoms beyond established risk factors (e.g., appearance‐RS, body‐ideal internalization), other unaccounted factors may play an important role. Previous research has demonstrated that participation in appearance‐focused behaviors mediated the relationship between photo‐based media use and body dissatisfaction (Seekis et al. 2020). Unlike in‐person interactions, videoconferencing allows immediate engagement in maladaptive safety behaviors, such as body checking (e.g., scrutinizing one's face on‐screen), avoidance (e.g., turning off the camera), or appearance‐enhancing actions (e.g., using face filters) (Jarman et al. 2023; Hart et al. 2022). While these behaviors temporarily alleviate appearance‐related distress, they may reinforce dysfunctional appearance beliefs and eating concerns (Nikodijevic et al. 2018; Solomun‐Krakus and Sabiston 2017). Hence, similar to photo‐based social media, videoconferencing may affect body image and eating concerns indirectly through these safety behaviors.

Alternatively, individuals with pre‐existing body image concerns may prefer videoconferencing over in‐person interactions for work/study (e.g., enrolling in virtual university classes or lectures) due to increased self‐presentation control. Additionally, as videoconferencing environments focus on facial appearance (e.g., self‐view typically displays only the face and upper body) this may limit its broader effects on body image and eating concerns, explaining the modest associations. Indeed, research shows 42.4% of users reported new facial appearance concerns during video calls (Pikoos et al. 2021). Given emerging evidence linking EDs to facial appearance concerns, including dissatisfaction, lower perceived attractiveness, and greater perceived adiposity (Portingale et al. 2025), further exploration into the adverse effects of videoconferencing use on facial perception is needed.

### 4.2 Differential Associations Between Videoconferencing Usage Frequency and Disordered Eating Symptom Types

Our findings also revealed that higher‐frequency videoconferencing usage was associated with greater dieting and oral control DE symptoms but was unrelated to bulimia/food preoccupation symptoms. This pattern partially aligns with previous research that found no significant association between videoconferencing usage and binge eating symptoms (Gullo and Walker 2021), while diverging from EMA findings of null effects on state‐level urges for both restrictive eating and overeating (Portingale, Kenny, et al. 2024). The differential, yet modest findings in our study may be better understood by considering the contextual and psychological mechanisms.

First, as videoconferencing inherently places others' appearances in constant view, frequent videoconferencing may increase exposure to sociocultural appearance ideals (e.g., thinness, muscularity) and heighten upwards appearance‐based social comparisons. Such comparisons are often associated with restrictive eating behaviors as a means of achieving these ideals (Portingale, Girardin, et al. 2024). In the specific context of work or study environments, appearance comparisons may still arise, but the intensity of these comparisons may be somewhat mitigated compared to videoconferencing for peer or social relationships, which typically carry greater appearance‐related social pressures (Fardouly and Vartanian 2015). This distinction may help to explain the modest associations observed in the current study.

Second, self‐viewing during videoconferencing has been associated with heightened anxiety (Gullo and Walker 2021) and negative emotional states have been linked to restrictive eating (Engel et al. 2013). The act of frequently seeing oneself on screen may exacerbate self‐critical thoughts or appearance‐related distress, which may lead some individuals to engage in dieting and oral control behaviors. These behaviors are believed to serve as emotional avoidance strategies, allowing individuals to temporarily escape from or suppress difficult emotions by focusing intensely on food and weight (Engel et al. 2013; Wildes et al. 2010). This coping mechanism could explain why videoconferencing usage was uniquely associated dieting and oral control symptoms, but not binge eating symptoms, which are often driven by other factors such as impulsivity.

### 4.3 Videoconferencing Usage Frequency Is Unrelated to Depressive Symptoms

Contrary to some cross‐sectional studies (Choukas‐Bradley et al. 2022; Gullo and Walker 2021), we found no significant association between videoconferencing usage frequency and depressive symptoms. This result aligns with previous EMA research, which found no relationship between videoconferencing usage and mood at the state level (Portingale, Kenny, et al. 2024). One possible explanation for this null effect is that videoconferencing environments, being inherently appearance‐focused (Jarman et al. 2023; Pfund et al. 2020; Pikoos et al. 2021), may have a more specific impact on appearance‐related concerns than broader mood states, particularly for frequent users. Our findings also support suggestions that any potential impact of videoconferencing on mood may be transient and only detected through state‐based assessments conducted at short time intervals, which capture fluctuations in mood (Portingale, Kenny, et al. 2024). Nonetheless, our predominantly young adult sample may be more resilient to the mood effects of videoconferencing, possibly due to greater familiarity with digital communication (Villanti et al. 2017). Future research targeting individuals diagnosed with or at high risk for major depressive disorder may be more adequately powered to detect potential effects.

### 4.4 Limited Moderating Effects of Videoconferencing Usage Frequency

Our analysis revealed only three significant moderating effect of videoconferencing usage frequency, but their explanatory power for this effect was limited, warranting cautious interpretation. Higher (vs. lower) frequency videoconferencing usage weakened the positive relationship between appearance‐RS and DE symptoms, specifically oral control. Higher frequency usage also strengthened the positive relationship between self‐objectification and bullimia/food preoccupation DE symptoms. This pattern indicates that individuals with a tendency to self‐objectify might be more vulnerable to developing bullimic eating behaviours. Interestingly, this pattern also indicates that individuals with appearance‐RS might be less vulnerable to developing restrictive eating behaviors in contexts where self‐presentation concerns are salient, such as videoconferencing. This finding broadly contrasts with prior research, whereby perceived social rejection was associated with greater restrictive eating behaviors (Beekman et al. 2017) and appearance‐RS was indirectly associated with greater restrictive eating behaviors via body dissatisfaction (Kimball et al. 2019). However, the modest effects sizes and absence of significant moderation effects for most relationships examined suggests that the associations between ED‐related psychological factors and symptoms are largely consistent between videoconferencing usage frequency groups. These findings may be attributed to the community‐based sample, which potentially lacks the clinical severity necessary to detect true effects. Future research using clinical ED and major depressive disorder samples is warranted.

### 4.5 Limitations and Future Directions

Several methodological limitations warrant consideration. First, our sample predominantly comprised young Asian women in Australia during COVID‐19, limiting generalizability. Future research should examine these relationships across diverse populations and cultural contexts.

Second, our reliance on self‐reported data may introduce recall bias (Heron and Smyth 2013; Smyth and Stone 2003), particularly for ED‐related symptoms (Svaldi et al. 2010) and videoconferencing usage. Future studies should incorporate passive platform data or EMA designs to capture objective usage and real‐time symptoms.

Third, our cross‐sectional design precludes causal inferences and potential habituation effects across the pandemic period (2020–2022). Drawing from exposure theory and therapeutic evidence that mirrors or videos effectively improve body image and eating concerns (Reilly et al. 2017; Trentowska et al. 2017), initial videoconferencing‐related appearance concerns may naturally attenuate through repeated exposure. Longitudinal research should examine temporal adaptation patterns, including how video‐enhancement features and pre‐existing body image concerns influence habituation trajectories.

Fourth, while our binary classification of videoconferencing usage was pragmatically justified, future research should develop standardized measures capturing multifaceted frequency categorizations (e.g., low, moderate, high) and engagement aspects (e.g., self‐view preferences, enhancement feature usage) and examine how different contexts (e.g., peer interactions, online dating) uniquely influence body image, eating concern, and mood.

Fifth, the centrality of facial appearance and availability of appearance enhancing filters within videoconferncing contexts is concerning. That is, given mounting evidence that facial beauty filter usage is linked to increased facial dissatisfaction (Rowland 2022) and the established link between facial appearance concerns (e.g., dissatisfaction, lower perceived attractiveness) and ED risk (Portingale et al. 2025). Future research on videoconferencing and EDs should examine facial appearance concerns as an outcome and the mediating effect of appearance‐enhancement practices on such relationship.

Finally, future research would benefit from advanced data quality checks (e.g., inconsistent responding across related items) and specific bot detection methods to safeguard against automated responses, particularly for large‐scale online data collection.

### 4.6 Implications

Insofar as future research can replicate and extend current findings using advanced methodologies and more nuanced measures, these insights could inform practices to protect against body image and eating concerns among videoconferencing users. Potential interventions include (i) collaborations between videoconferencing platform developers and ED clinicians to create guidance on using features like “gallery view" or hiding self‐view; (ii) making the “hide self‐view" option more accessible within platforms; (iii) encouraging clinicians, educators, and caregivers to help young adults focus on body functionality rather than appearance; and (iv) implementing behavioral experiments to reduce avoidance and safety behaviors while increasing adaptive anxiety regulation strategies like self‐compassion (Carels et al. 2021).

## 5 Conclusion

This study demonstrates unique associations between frequent videoconferencing use for work/study and specific ED‐related symptoms, particularly body dissatisfaction and dietary restraint behaviors. However, these relationships were modest, and videoconferencing explained minimal variance beyond established psychological risk factors. Videoconferencing frequency was unrelated to depressive symptoms and bulimia/food preoccupation, suggesting specificity in its associations. Moreover, videoconferencing usage frequency generally failed to moderate the influence of ED‐related psychological risk factors on ED symptoms, with a few modest exceptions. These findings indicate that while videoconferencing may play a unique role in certain ED‐related symptoms, its influence appears more nuanced and limited than initially theorized. Future research should test these relationships in clinical ED populations, whilst examining more specific outcomes (e.g., facial appearance concerns) and different videoconferencing usage contexts (e.g., social interactions). As videoconferencing remains a prominent communication mode, continued investigation of these nuanced relationships is paramount.

## Author Contributions

**Jade Portingale:** conceptualization, data curation, formal analyses, methodology, investigation, writing – Original draft, writing – review & editing. **Isabel Krug:** conceptualization, methodology, project administration, supervision, writing – review & editing.

## Conflicts of Interest

The authors declare no conflict of interest.

## Supporting information

Table S1

## Data Availability

The data that support the findings of this study are available from the corresponding author upon reasonable request.
