# Supplementary material for: Virtual (Self) Reflection: Frequent Videoconferencing Usage Is Uniquely Associated With Body Dissatisfaction and Dietary Restraint Symptoms Among Adults
Source: Eur Eat Disord Rev. 2025 Mar 21;33(5):904–17. doi: 10.1002/erv.3191 (PMC12319137; doi:10.1002/erv.3191)
Supplement: Supplementary file 1 — Table S1 [file ERV-33-904-s001.docx]

**Supplementary Material: Table A**

*Hierarchical Regression Analyses Predicting Body Dissatisfaction, Disordered Eating Symptoms, and Depressive Symptoms from Continuous Videoconferencing Usage Frequency*

|  | | Body dissatisfaction | | | DE (overall) | | | DE (oral control) | | | | DE (bulimia/food preoccupation) | | | DE (dieting) | | | Depressive symptoms | | |  |
| --- | --- | --- | --- | --- | --- | --- | --- | --- | --- | --- | --- | --- | --- | --- | --- | --- | --- | --- | --- | --- | --- |
|  | | *β* | *t* | *p* | *β* | *t* | *p* | *β* | | *t* | *p* | *β* | *t* | *p* | *β* | *t* | *p* | *β* | *t* | *p* | |
| ***Step 1*** | |  |  |  |  |  |  |  | |  |  |  |  |  |  |  |  |  |  |  | |
| Age | | -.014 | -.628 | .530 | -.015 | -.658 | .510 | **-.054** | | **-2.274** | **.023** | .002 | .067 | .947 | .000 | .021 | .983 | **-.056** | **-2.343** | **.019** | |
| BMI | | .013 | .549 | .583 | .012 | .506 | .613 | .008 | | .332 | .740 | -.013 | -.570 | .569 | .016 | .686 | .493 | .003 | .106 | .916 | |
| Lifetime eating disorder diagnosis: no | | -.107 | -4.646 | <**.001** | -.268 | -11.687 | <**.001** | -.185 | | -7.780 | <.**00**1 | -.281 | -12.267 | <**.001** | -.227 | -9.794 | **<.001** | -.098 | -4.105 | **<.001** | |
| Gender: male | | -.109 | -1.180 | .238 | .074 | .796 | .426 | .014 | | .151 | .880 | .158 | 1.713 | .087 | .050 | .531 | .596 | -.361 | -3.756 | **<.001** | |
| Gender: female | | .174 | 1.885 | .060 | **.219** | **2.371** | **.018** | .043 | | .453 | .650 | .283 | 3.066 | .**002** | **.203** | **2.176** | **.030** | -.295 | -3.071 | **.002** | |
|  | |  |  |  |  |  |  |  | |  |  |  |  |  |  |  |  |  |  |  | |
|  | | R² = .097, ΔR² = .097, *F*(5, 1733) = 37.400, *p* **< .001** | | | R² = .101, ΔR² = .101, *F*(5, 1733) = 38.893, *p* < **.001** | | | R² = .039, ΔR² = .039, *F*(5, 1733) = 13.939, *p* < **.001** | | | | R² = .102, ΔR² = .102, *F*(5, 1733) = 39.553, *p* < **.001** | | | R² = .082, ΔR² = .082, *F*(5, 1733) = 30.930, *p* < **.001** | | | R² = .027, ΔR² = .027, *F*(5, 1733) = 9.436, *p* < **.001** | | | |
| ***Step 2*** | |  |  |  |  |  |  |  |  | |  |  |  |  |  |  |  |  |  |  | |
| Appearance-RS | | .370 | 19.167 | **<.001** | .283 | 12.861 | **<.001** | .122 | 4.719 | | **<.001** | .251 | 10.671 | **<.001** | .280 | 12.903 | **<.001** | .388 | 16.216 | **<.001** | |
| Body-ideal internalisation | | .385 | 20.409 | **<.001** | .290 | 13.517 | **<.001** | .084 | 3.336 | | **<.001** | .183 | 7.998 | **<.001** | .343 | 16.232 | **<.001** | .077 | 3.312 | **<.001** | |
| Self-objectification | | .013 | .723 | .469 | .024 | 1.162 | .245 | .035 | 1.473 | | .141 | .013 | .613 | .540 | .018 | .881 | .378 | .009 | .389 | .697 | |
|  | |  |  |  |  |  |  |  |  | |  |  |  |  |  |  |  |  |  |  | |
|  | | R² = .479, ΔR² = .382, *F*(3, 1730) = 423.185, *p* < **.001** | | | R² = .324, ΔR² = .223, *F*(3, 1730) = 190.650, *p* < **.001** | | | R² = .071, ΔR² = .032, *F*(3, 1730) = 20.019, *p* **< .001** | | | | R² = .231, ΔR² = .128, *F*(3, 1730) = 96.191, *p* **< .001** | | | R² = .345, ΔR² = .263, *F*(3, 1730) = 231.557, *p* **< .001** | | | R² = .201, ΔR² = .174, *F*(3, 1730) = 125.594, *p* **< .001** | | | |
| ***Step 3*** | |  |  |  |  |  |  |  | |  |  |  |  |  |  |  |  |  |  |  | |
| Videoconferencing usage frequency | | .042 | 2.444 | **.015** | .073 | 3.671 | **<.001** | .075 | | 3.228 | **.001** | .050 | 2.355 | **.019** | .063 | 3.230 | **.001** | .025 | 1.169 | .243 | |
|  | | R² = .481, ΔR² = .002, *F* (8, 1730) = 200.588, *p* < **.001** | | | R² = .329, ΔR² = .005, *F*(8, 1730) = 106.067, *p* < **.001** | | | R² = .076, ΔR² = .005, *F*(8, 1730) = 17.906, *p* **< .001** | | | | R² = .231, ΔR² = .000, *F*(8, 1730) = 65.061, *p* **< .001** | | | R² = .349, ΔR² = .004, *F*(8, 1730) = 115.759, *p* **< .001** | | | R² = .191, ΔR² = .002, *F*(8, 1730) = 50.967, *p* **< .001** | | | |
| ***Step 4*** | |  |  |  |  |  |  |  | |  |  |  |  |  |  |  |  |  |  |  | |
| Videoconferencing usage frequency* self-objectification | | -.002 | -.052 | .958 | .094 | 1.911 | .056 | .094 | | 1.616 | .106 | **.106** | **2.003** | **.045** | .066 | 1.358 | .175 | .022 | .396 | .692 | |
| Videoconferencing usage frequency*appearance-RS | | -.100 | -1.642 | .101 | -.091 | -1.312 | .190 | -.213 | | -2.620 | **.009** | **-.080** | **-1.075** | **.283** | -.039 | -.574 | .566 | -.062 | -.816 | .414 | |
| Videoconferencing usage frequency* body-ideal internalisation | | .131 | 1.581 | .114 | -.072 | -.764 | .445 | .027 | | .242 | .809 | **-.091** | **-.903** | **.367** | -.078 | -.843 | .399 | .022 | .215 | .830 | |
|  | | R² = .482, ΔR² = .001, *F* (11, 1727) = 146.336, *p* < **.001** | | | R² = .331, ΔR² = .002, *F*(11, 1727) = 77.767, *p* < **.001** | | | R² = .081, ΔR² = .005, *F*(11, 1727) = 13.806, *p* **< .001** | | | | R² = .234, ΔR² = .003, *F*(11, 1727) = 47.907, *p* **< .001** | | | R² = .350, ΔR² = .001, *F*(11, 1727) = 84.438, *p* **< .001** | | | R² = .191, ΔR² = .001, *F*(11, 1727) = 37.082, *p* **< .001** | | | |
|  |  |  |  |  |  |  |  |  |  |  |  |  |  |  |  |  |  |  |  |  |  |

*Note*. Model 1 includes demographic and clinical covariates; Model 2 adds ED-related psychological factors; Model 3 adds videoconferencing frequency (continuous); Model 4 adds interaction terms. DE = disordered eating; BMI = body mass index. Reference categories assigned a value of 0: Gender = Other/prefer not to say; Eating disorder = lifetime diagnosis (yes). Significant results are bolded: p < .05 for body dissatisfaction and depressive symptoms; p < .0125 for disordered eating symptoms (Bonferroni-corrected for the four DE measures).
